# Plant hormone-targeted metabolomics revealed the temperature regulation of tuber growth and secondary metabolites in *Gastrodia elata* f. *glauca*

**DOI:** 10.3389/fpls.2026.1823326

**Published:** 2026-05-01

**Authors:** Xinlei Xu, Daichuan Pan, Guosheng Zhu, Xu Zeng, Shunxing Guo

**Affiliations:** 1 StateKey Laboratory of Bioactive Substance and Function of Natural Medicines, Institute of Medicinal Plant Development, Chinese Academy of Medical Sciences and Peking Union Medical College, Beijing, China; 2 Biotechnology Institute of Guizhou, Guizhou Academy of Agricultural Sciences, Guiyang, China

**Keywords:** *Gastrodia elata* f. *glauca*, phytometabolite, plant hormones, secondary metabolites, temperature

## Abstract

**Introduction:**

*Gastrodia elata* f. *glauca* represents a superior cultivar of *G. elata* with considerable commercial value and broad market acceptance. The growth of its tubers and the accumulation of bioactive secondary metabolites in this variant are strongly affected by environmental temperature.

**Methods:**

In this study, UPLC and targeted phytohormone metabolomics were used to investigate the effects of different temperatures on growth and metabolic processes in *G. elata* f. *glauca*.

**Results:**

The results showed that biomass accumulation was greatest at 20 °C. Meanwhile, the contents of gastrodin, p-hydroxybenzyl alcohol, parishin A, parishin B, and parishin C were maintained at relatively elevated levels at 15 °C, whereas parishin E exhibited higher accumulation at 25 °C. Targeted metabolomics and correlation analysis indicated that increasing temperature upregulated auxin- and gibberellin-related metabolites, which may promote tuber growth, whereas lower temperature downregulated indole-related metabolites. In contrast, moderate plant hormone levels were more conducive to the accumulation of bioactive constituents.

**Discussion:**

These results suggest a close association among temperature, plant hormones, growth, and bioactive secondary metabolites accumulation in *G. elata* f. *glauca*. They provide a basis for further investigation of temperature-related metabolic responses and may help optimize cultivation practices for improved yield and quality.

## 1 Introduction

*Gastrodia elata* Blume is a myco-heterotrophic herb belonging to the Orchidaceae family (Zhang et al., 2022). In recent decades, its tubers have gained recognition as an important edible and medicinal resource, particularly in variants such as *G. elata* f. *glauca* with high market acceptance. This variant is renowned for its diverse pharmacological activities, including the treatment of cardiovascular, neurological, and metabolic diseases (Huang et al., 2021; Yang and Tsai, 2022; Peng et al., 2023; Xu et al., 2023). *G. elata* contains various active compounds, including gastrodin, p-hydroxybenzyl alcohol, parishin A, parishin B, parishin C, and parishin E (Wang and Shahid, 2023; Zeng et al., 2023). These compounds are primarily biosynthesized through the phenylalanine pathway (Fu et al., 2024; Wei et al., 2024). Moreover, *Gastrodia elata* is rich in diverse nutrients, including proteins, starch, polysaccharides, eight essential amino acids, and selenium (Li X. et al., 2024). Therefore, *Gastrodia elata* has broad potential for development and application.

According to the Flora of China, *G. elata* comprises five primary varieties, of which *G. elata* f. *elata*, *G. elata* f. *glauca*, and *G. elata* f. *viridis* are the main forms currently under cultivation (Zhou and Chen, 1983). Among these, *G. elata* f. *glauca* commands the highest market price because of its superior quality and more favorable taste profile (Cui et al., 2023; Zhong et al., 2025). *G. elata* f. *glauca* is primarily distributed in high-altitude mountainous regions (>1,500 m) (Zou et al., 2023), where temperature strongly influences its entire life cycle, including seed germination (Luo et al., 2019), tuber development (Li et al., 2020), and bioactive components accumulation (Jiang et al., 2025). Seed germination is particularly sensitive to temperature, with optimal performance at 16 °C compared with 12 °C and 20 °C (Diao and Guo, 2025). Zhu’s study showed that climatic factors had the strongest correlation with *G. elata* quality (Zhu et al., 2023). The total contents of gastrodin, p-hydroxybenzyl alcohol, parishin A, parishin B, parishin C, and parishin were positively correlated with annual average temperature. Despite known effects of temperature on germination and bioactive compounds in *G. elata* f. *glauca*, the temperature-mediated variation in endogenous hormones and its potential link to secondary metabolite accumulation remain largely unclear.

Plant hormones are small organic molecules synthesized within plants that function as key regulators of growth, secondary metabolite accumulation, and environmental stress responses (Du et al., 2013; Ali et al., 2024). Moderately elevated temperatures enhance auxin signaling in *Arabidopsis* roots, promoting cell division and elongation to increase primary root length, while cytokinin levels and signaling are adjusted to balance the growth response (Jacob et al., 2025). Low temperatures induce adjustments in cytokinin and auxin levels in rice, which help maintain root and shoot development, while promoting abscisic acid accumulation to regulate osmotic protection and modulate gene expression for adaptive physiological responses (Yang, 2022). Low temperatures also induce elevated levels of abscisic acid and jasmonic acid in *Salvia miltiorrhiza*, coupled with modulated auxin and cytokinin signaling. These hormonal changes upregulate key genes in the phenylpropanoid and terpenoid biosynthetic pathways, leading to enhanced accumulation of phenolic acids and tanshinones, thereby improving bioactive compound contents and medicinal quality (He et al., 2023; Li H. et al., 2024).

In this study, *G. elata* f. *glauca* was used as the experimental material. Tubers were cultivated under five different temperatures regimes for 120, 160, and 200 days. The contents of six bioactive constituents were determined by UPLC. In addition, targeted phytohormone metabolomics was performed on samples collected after 160 days at 10 °C, 15 °C, and 20 °C to quantify 39 metabolites belonging to eight major phytohormone classes. Correlation analysis was used to investigate the relationships among temperature, biomass accumulation, bioactive compounds, and plant hormones. Therefore, this study investigated the effects of different temperatures on the accumulation of bioactive secondary metabolites and endogenous phytohormone profiles in *G. elata* f. *glauca* tubers, aiming to elucidate their potential regulatory relationships and provide a scientific basis for optimized cultivation.

## 2 Materials and methods

### 2.1 Plant material, symbiotic cultivation, and temperature treatment conditions

Tubers of *G. elata* f. *glauca* were collected from Mianyang, Sichuan Province, China. The *Armillaria* strain used in this study was isolated and purified from a commercial *Armillaria* sp. strain (designated as Ar6, provided by Chunguang Fungiculture Development Co., Ltd.) which was shown an effective symbiosis with *G. elata* in our preliminary experiments (Diao et al, 2026). To obtain solid spawn for subsequent experiments, the strain was co-cultured with small *Cyclobalanopsis glauca* wood sticks (5 cm × 2 cm), a standard practice for *G. elata* cultivation (Xu, 1993). The cultivation method was modified from Zhou’s method (Zhou et al., 2023): for symbiotic cultivation under controlled conditions, fresh tubers of *G. elata* f. *glauca*, the solid spawn, and sand were layered into sterile culture bottles (1000 mL capacity). The assembled bottles were incubated in complete darkness at temperatures of 4, 10, 15, 20, and 25 °C in a programmable incubator with 40%-50% relative humidity and good ventilation. Tuber samples were harvested at 120, 160, and 200 days of incubation for subsequent analyses, with each temperature treatment replicated independently three independent biological replicates. The weight of *G. elata* f. *glauca* under different cultivation temperatures and durations was measured, and a growth index was used to represent its biomass (Nadzirah Abd Malik et al., 2022).


$$Growth\;index=\frac{final\;fresh\;weight-initial\;fresh\;weight}{initial\;fresh\;weight}*100\%$$

### 2.2 Determination of gastrodin, p -hydroxybenzyl alcohol, and parishins in *G. elata* f. *glauca* tubers

The extraction and quantification of gastrodin, p-hydroxybenzyl alcohol, parishin A, parishin B, parishin C, and parishin E were performed according to the Chinese Pharmacopoeia (Commission, 2020) and a previous report (Chen et al., 2019). An accurately weighed 0.5 g powder was placed in a 5 mL volumetric flask and extracted with 50% (v/v) methanol. After ultrasonic extraction for 60 min, additional 50% methanol was added to compensate for weight loss. Following filtration, 2 mL of the filtrate was accurately transferred to a 10 mL volumetric flask and diluted to volume with 3% (v/v) acetonitrile. The resulting solution was filtered through a 0.22 μm membrane before UPLC analysis.

UPLC analysis employed a mobile phase consisting of acetonitrile (A) and 0.05% (v/v) aqueous phosphoric acid (B). The gradient elution program was as follows: 0 min~8.5 min, 3% A; 8.5 min~9 min, 12% A; 9 min~13min, 18% A. The flow rate of 0.3 mL/min, an injection volume of 5 μL, and a detection wavelength of 220 nm were used. The column temperature was maintained at 40 °C.

### 2.3 Metabolomics analysis of targeted phytohormones

Targeted metabolomic profiling was carried out by a commercial service company (Biozeron, Shanghai). LC-MS was used to detect the concentration series of standard solution. The ratio of concentration of standard to internal standard as abscissa, and the ratio of peak area of standard to internal standard as ordinate to investigate the linearity of standard solution. The correlation coefficient (r) > 0.99 of each metabolite were the necessary condition. The limit of quantification (LOQ) was determined using the signal-to-noise ratio (S/N) method by comparing the signal from standard solutions with that of the blank matrix. Generally, when the S/N = 10:1, the corresponding concentration is the LOQ.

The samples were ground into powder in liquid nitrogen, and then added to water by well vortexing as the diluted sample. Then 100 μL of them were taken respectively and homogenized with 400 μL of 50% acetonitrile, which contained mixed internal standards, and extracted at 4 °C. The extracts were centrifuged at 12,000 rpm for 10 min. The supernatant (300 μL) was passed through HLB sorbent to obtain the first flow-through fraction and then eluted with 500 μL of 30% acetonitrile to obtain the second flow-through fraction. The two fractions were combined, mixed well, and injected into the LC-MS/MS system for analysis.

An ultra-high performance liquid chromatography coupled to tandem mass spectrometry (UHPLC-MS/MS) system was used to quantify phytohormones. The mobile phase, consisting of 0.01% formic acid in water (solvent A) and 0.01% formic acid in acetonitrile (solvent B), was delivered at a flow rate of 0.30 mL/min. The solvent gradient was set as follows: initial 10% B, 1min; 10-50% B, 3min; 50-65% B, 4 min; 65-70% B, 6min; 70-100% B, 7min; 100-10% B, 9.1min; 10% B, 12min.

The mass spectrometer was operated in multiple reaction mode (MRM). Parameters were as follows: IonSpray Voltage (Negative mode: -4500 V, Positive mode: 4500V), Curtain Gas (35 psi), Ion Source Temp (550 °C), Ion Source Gas of 1 and 2 (60 psi).

### 2.4 Statistical analysis

All experiments were carried out in three independent biological replicates. Statistical analyses and correlation analysis were conducted using SPSS version 27.0 for one-way ANOVA statistical analysis and Bonferroni method for multiple comparisons, with differences among temperature treatment groups considered statistically significant at p< 0.05. GraphPad Prism 10.1.2 was used for graphical plotting. For the targeted phytohormones, principal components analysis (PCA) was used to reflect the overall expression difference among the groups and the variation degree among the samples in the groups. We applied univariate analysis (t-test) to calculate the statistical significance (P-value). The metabolites with VIP > 1 and P-value< 0.05 and fold change ≥2 or FC ≤ 0.5 were considered to be differential metabolites. The functions of the differential metabolites and metabolic pathways were studied using the KEGG database.

## 3 Results

### 3.1 Effect of temperature on the biomass

**Figure 1:**
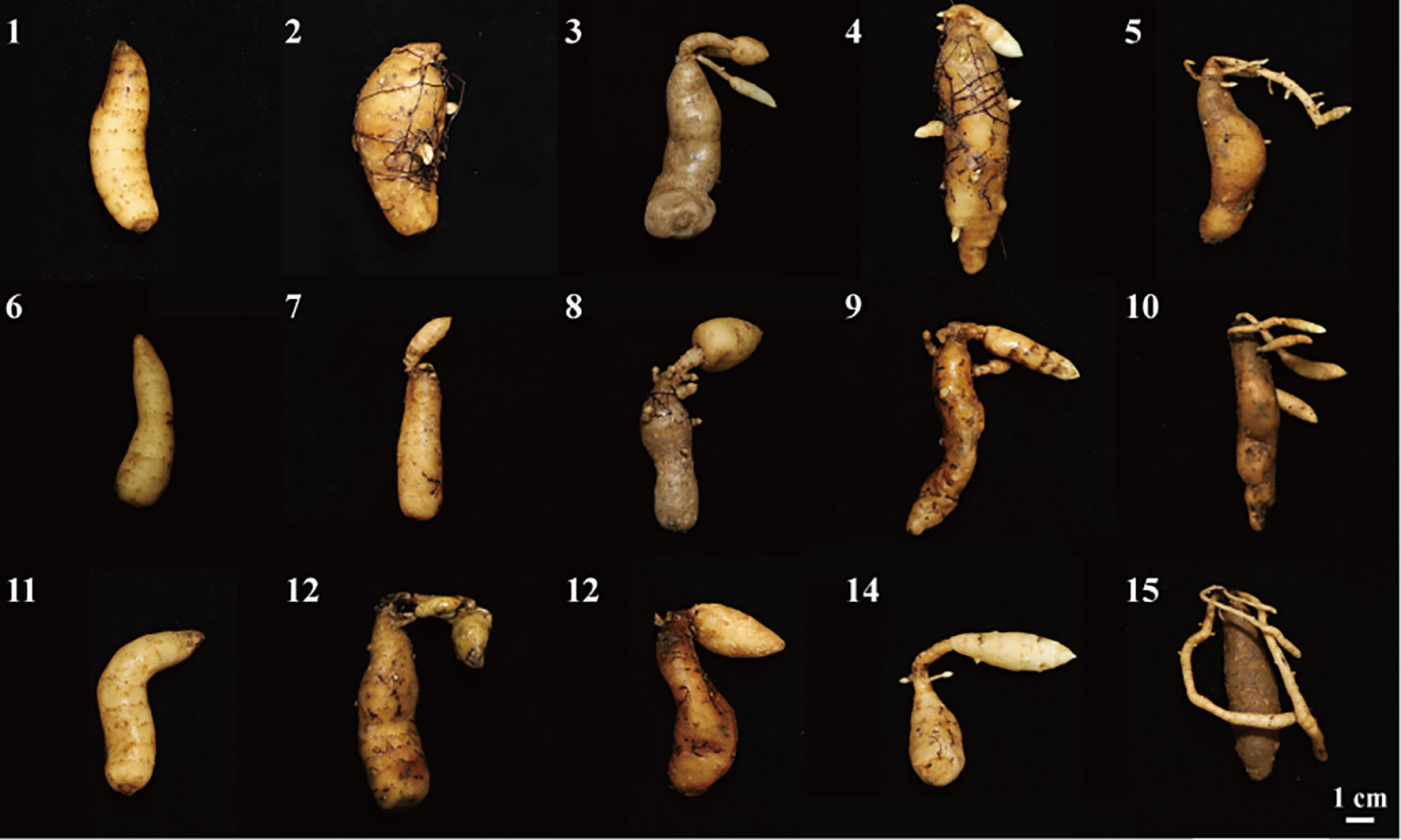
The growth status of *G. elata* f. *glauca*. 1-5: Growth status of *G. elata* f. *glauca* cultured at 4 °C, 10 °C, 15 °C, 20 °C, and 25 °C for 120 days; 6-10: Growth status of *G. elata* f. *glauca* cultured at 4 °C, 10 °C, 15 °C, 20 °C, and 25 °C for 160 days; 11-12: Growth status of *G. elata* f. *glauca* cultured at 4 °C, 10 °C, 15 °C, 20 °C, and 25 °C for 200 days.

As shown in **Figure 1**, the tubers of *G. elata* f. *glauca* grew slowly after 120 days cultivation. At 160 days of cultivation, growth of the newly formed tubers was observed under 15-25 °C conditions. As cultivation continued, the newly formed tubers enlarged further. The newly formed tubers were shorter and more rounded at 15 °C and 20 °C, whereas those grown at 25 °C were relatively more slender and elongated.

**Figure 2:**
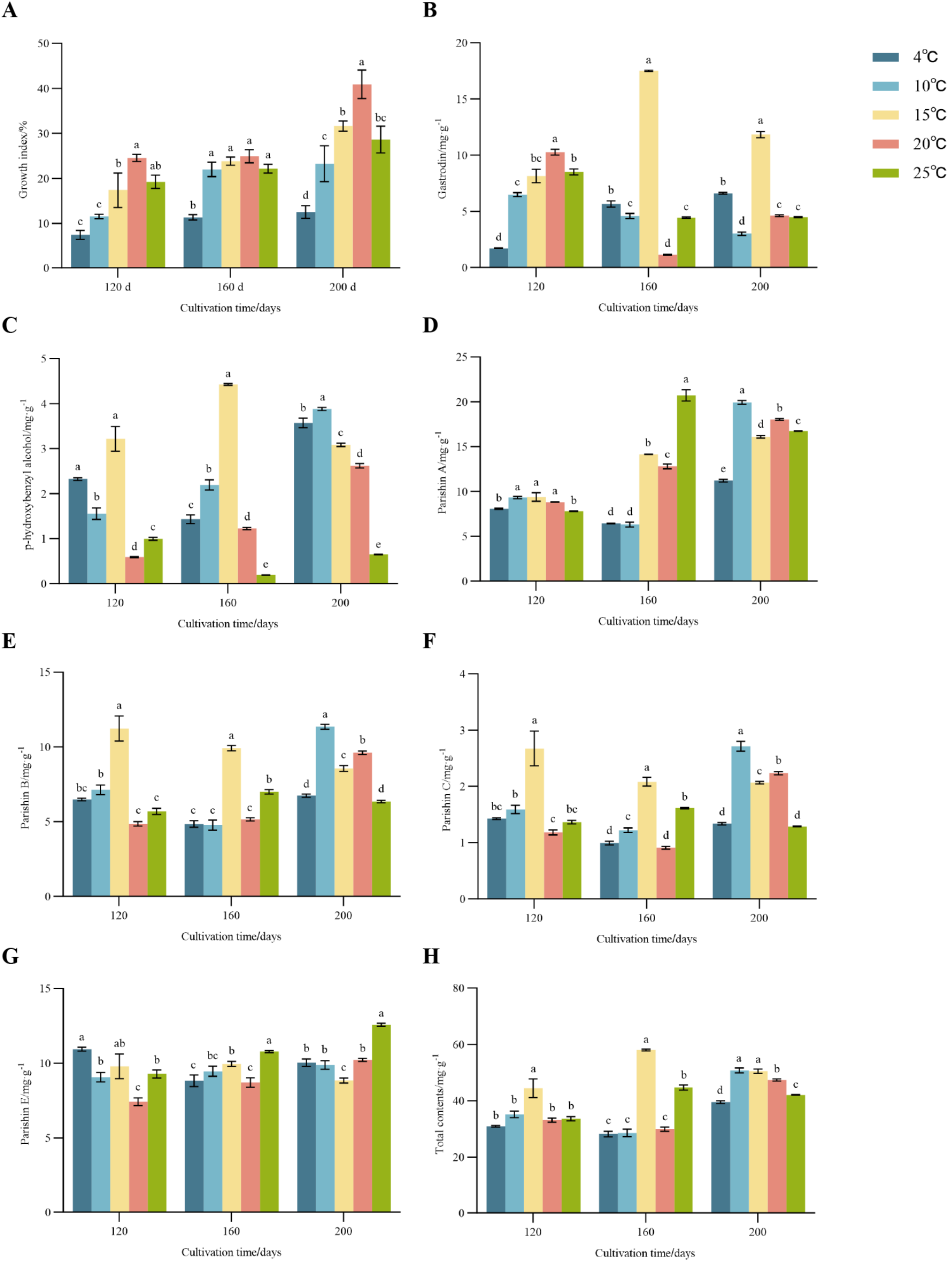
The growth index and contents of the 6 bioactive constituents. The growth index **(A)** and the content of Gastrodin **(B)**, p-hydroxybenzyl alcohol **(C)**, parishin A **(D)**, parishin B **(E)**, parishin C **(F)**, and parishin E **(G)** and total contents of the 6 bioactive constituents **(H)**. Values are average with their standard deviations (n=3) with three biological replicates. Different lowercase represents a significant difference in temperature among the same cultivation period (p< 0.05).

Temperature significantly influenced the biomass accumulation of *Gastrodia elata* f. *glauca* during the growth period. As shown in **Figure 2A**, the growth index exhibited a clear unimodal response to temperature, increasing initially and then decreasing with rising temperature, with the maximum value observed at 20 °C. The growth index at 4 °C was consistently low. At 120 days, the growth index at 20 °C reached 25.56%, which was significantly higher than that of other temperature treatments (p< 0.05). At 160 days, the growth index at 20 °C was 24.93%, with no significant difference from the values at 10 °C and 25 °C. This result suggests that *G. elata* f. *glauca* had entered a relatively stable growth phase at this time point. At 200 days, the growth index of *G. elata* f. *glauca* at 20 °C reached 40.92% (p< 0.05).

### 3.2 Content of bioactive compounds

#### 3.2.1 Gastrodin and p-hydroxybenzyl alcohol

With increasing temperature, gastrodin content in *G. elata* f. *glauca* first increased and then decreased (**Figure 2B**). At 120 days, gastrodin reached a maximum of 10.26 mg·g^-1^ at 20 °C (p< 0.05). Notably, at 160 days, the content at 15 °C was approximately 15-fold higher than that at 20 °C. By 200 days, the content at 15 °C had decreased to approximately 68% of the value recorded at 160 days. P-hydroxybenzyl alcohol content in *G. elata* f. *glauca* showed a similar pattern, increasing initially with rising temperature and then declining at higher temperatures (**Figure 2C**). At 120 days, relatively high contents were observed at 4 °C (2.32 mg·g^-1^) and 15 °C (3.22 mg·g^-1^). At 160 days, p-hydroxybenzyl alcohol reached its maximum at 15 °C (4.43 mg·g^-1^) (p< 0.05). At 200 days, the highest content was recorded at 10 °C (3.88 mg·g^-1^, p< 0.05).

These observations indicate that temperatures around 15 °C favor the accumulation of gastrodin and p-hydroxybenzyl alcohol in *G. elata* f. *glauca* tubers, particularly during the later growth stages (160–200 days), while higher temperatures, such as 20 °C, supports greater accumulation mainly in the early stage (120 days).

#### 3.2.2 Parishin A, parishin B, parishin C, and parishin E

Parishin A content in *G. elata* f. *glauca* increased continuously throughout the cultivation period (**Figure 2D**). At 120 days, no significant differences were observed among the 10-20 °C treatments, suggesting relatively stable accumulation during this cultivation stage. At 160 days, parishin A content peaked at 25 °C, reaching 20.72 mg·g^-1^ (p< 0.05). At 200 days, the highest parishin A content was observed at 10 °C (19.93 mg·g^-1^; p< 0.05).

Parishin B and C exhibited similar responses to cultivation time and temperature (**Figures 2E, F**). Their contents increased initially with rising temperature and then decreased at higher temperatures. At 120 days and 160 d days, both parishin B and C reached maximum levels at 15 °C and decreased significantly with further temperature elevation. At 200 days, the highest contents of parishin B and C were recorded at 10 °C, reaching 11.34 mgg and 2.71 mg·g^-1^, respectively (p< 0.05).

In contrast, parishin E showed a different pattern (**Figure 2G**). At 160 d and 200 days, parishin E content was highest at 25 °C, reaching 10.77 mg·g^-1^ and 12.57 mg·g^-1^, respectively (p<0.05).

Collectively, these results indicate that cultivation at 10 °C and 15 °C promotes the biosynthesis and accumulation of parishins A, B, and C, whereas parishin E accumulates to a higher level at 25 °C.

#### 3.2.3 Total contents of the six bioactive compounds

With increasing cultivation temperature, the total contents of the six bioactive compounds in *G. elata* f. *glauca* first increased and then decreased (**Figure 2H**). At 120 days and 160 days, the total contents reached their maximum at 15 °C, reaching 44.44 mg·g^-1^ and 58.01 mg·g^-1^, respectively (p<0.05). At 200 days, relatively high total contents were observed at 10 °C (50.78 mg·g^-1^) and 15 °C (50.48 mg·g^-1^).

### 3.3 The metabolome analysis

#### 3.3.1 Targeted metabolome analysis

**Figure 3:**
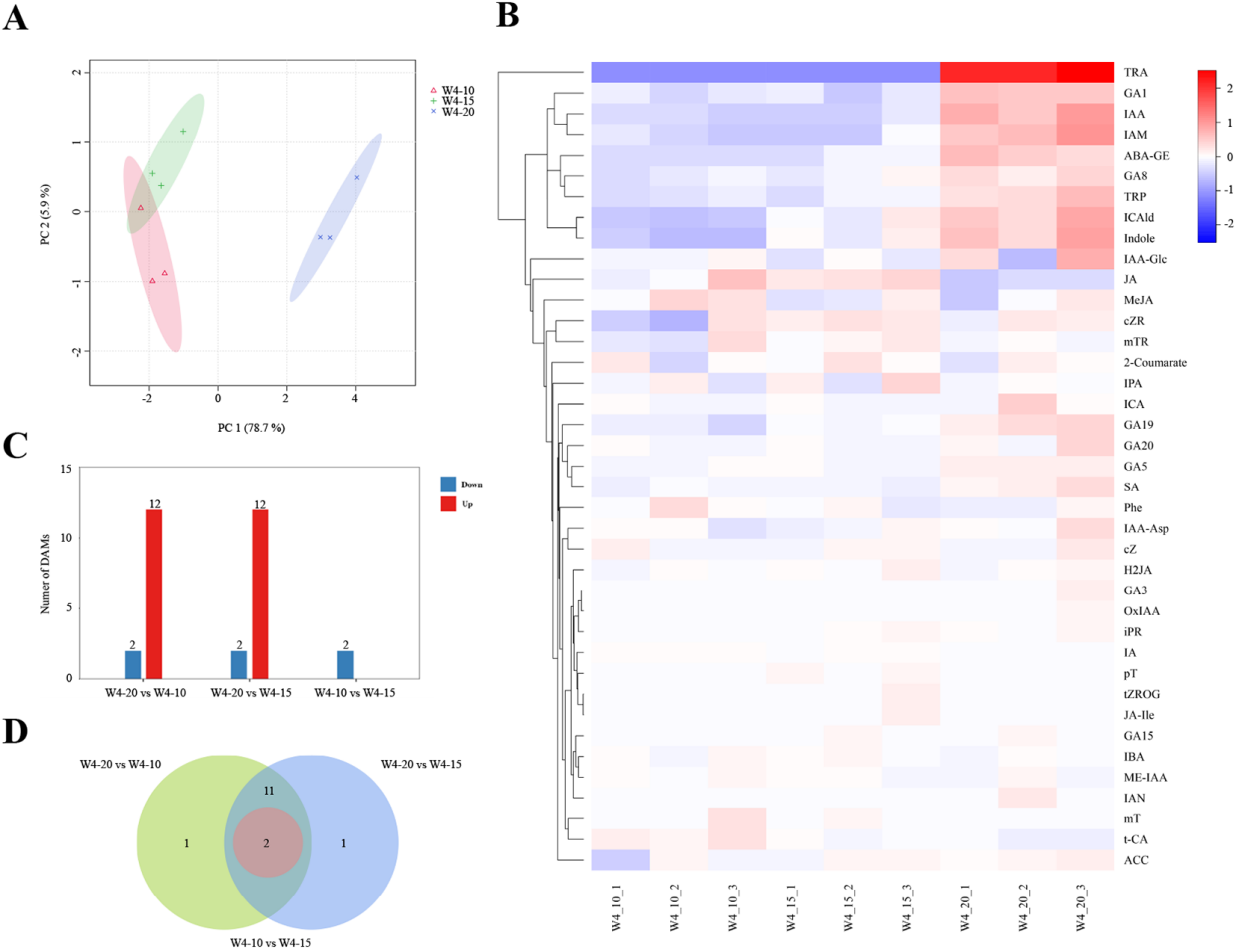
The metabolite analysis of *G. elata* f. *glauca* during different temperatures. PCA analysis of metabolites **(A)**. Heatmap of 39 endogenous phytohormones and hierarchical clustering analysis **(B)**. Numbers of DAMs **(C)**. DAMs Venn diagram **(D)**.

To further investigate the effects of temperature on phytohormone profiles in *G. elata* f. *glauca* tubers, targeted metabolome analysis of plant hormones was conducted using LC-ESI-MS/MS. According to the growth index and bioactive compounds data, samples were collected at 160 d under three temperature conditions: 10 °C (W4-10), 15 °C (W4-15), and 20 °C (W4-20). Multivariate statistical analysis revealed clear temperature-dependent differences in hormone profiles. In total, 39 metabolites belonging to eight classes were detected (**Table 1**). To further characterize phytohormone distribution patterns, principal component analysis (PCA) was performed (**Figure 3A**). In the PCA score plot, W4–20 samples were clearly separated from W4–10 and W4–15 samples, indicating a distinct phytohormone profile under the 20 °C condition.

**Table 1:** All metabolites and their classifications.

Name	Full name	Classifications
Indole	Indole	Auxin
ICAld	Indole-3-carboxaldehyde	Auxin
IAN	Indole-3-acetonitrile	Auxin
TRA	Tryptamine	Auxin
ICA	Indole-3-carboxylic acid	Auxin
IAM	Indol-3-acetamide	Auxin
IAA	Indole-3-acetic acid	Auxin
ME-IAA	Methyl indole-3-acetate	Auxin
IPA	3-Indolepropionic acid	Auxin
IBA	Indole-3-butyric acid	Auxin
TRP	Tryptophan	Auxin
IA	3-Indoleacrylic acid	Auxin
OxIAA	2-oxoindole-3-acetic acid	Auxin
IAA-Asp	IAA-aspartate	Auxin
IAA-Glc	1-O-indol-3-ylacetylglucose	Auxin
cZ	cis-Zeatin	Cytokinins
mT	meta-topolin	Cytokinins
iPR	Isopentenyl adenosine	Cytokinins
mTR	meta-topolin riboside	Cytokinins
tZROG	trans-zeatin riboside-O-glucoside	Cytokinins
pT	para-topolin	Cytokinins
cZR	cis-zeatin riboside	Cytokinins
GA15	Gibberellin A15	Gibberellins
GA5	Gibberellin A5	Gibberellins
GA20	Gibberellin A20	Gibberellins
GA3	Gibberellin A3	Gibberellins
GA1	Gibberellin A1	Gibberellins
GA19	Gibberellin A19	Gibberellins
GA8	Gibberellin A8	Gibberellins
MeJA	Methyl jasmonate	Jasmonic acid
JA-Ile	N-Jasimonic acid-Isoleucine	Jasmonic acid
JA	(+/-)-Jasmonic acid	Jasmonic acid
H2JA	Dihydrojasmonic acid	Jasmonic acid
ABA-GE	Abscisic acid glucose ester	Abscisic acid
ACC	1-aminocyclopropane-1-carboxylic acid	Ethylene
SA	Salicylic acid	Salicylic acid
t-CA	trans-Cinnamic acid	Others
2-Coumarate	2-Coumarate	Others
Phe	L-phenylalanine	Others

Significant temperature-related changes were observed in the levels of various phytohormones (**Figure 3B**). At 20 °C, the contents of several auxin-related compounds, including TRA, IAA, IAM, TRP, ICAld, indole, IAA-Glc, and ICA, increased markedly, together with gibberellins (GA1, GA8, GA19, GA20, and GA5), ABA-GE, and SA. In contrast, jasmonic acid-related compounds (JA and MeJA) showed significantly lower levels at 20 °C compared with 10 °C and 15 °C.

#### 3.3.2 Differentially accumulated metabolite analysis

Differential metabolites were identified based on variable importance in projection (VIP) values derived from the OPLS-DA model in combination with p-values from independent-samples t-tests. Metabolites meeting the criteria of |log2FC|≥1, VIP≥1 and P≤0.05 were defined as differential metabolites. The W4–20 vs. W4–15 and W4–20 vs. W4–10 comparisons shared 12 commonly upregulated metabolites, including indole, indole-3-carboxaldehyde, tryptamine, indol-3-acetamide, indole-3-acetic acid, tryptophan, gibberellin A5, gibberellin A1, gibberellin A19, gibberellin A8, salicylic acid and abscisic acid glucose ester (**Figure 3C**). Furthermore, each pairwise comparison identified 2 downregulated differential metabolites: 3-Indoleacrylic acid and trans-Cinnamic acid in W4–20 vs. W4-10, 3-indoleacrylic acid and (+/-)-jasmonic acid in W4–20 vs. W4-15, indole and indole-3-carboxaldehyde in W4–15 vs. W4-10. Notably, indole and indole-3-carboxaldehyde showed significant variation across all groups according to Venn analysis (**Figure 3D**). They were upregulated with increasing temperature.

#### 3.3.3 The functional analysis of DAMs

To further explore the biological functions of DAMs under different temperatures conditions in *G. elata* f. *glauca*, significantly differential metabolites were annotated and classified using KEGG pathways.

**Figure 4:**
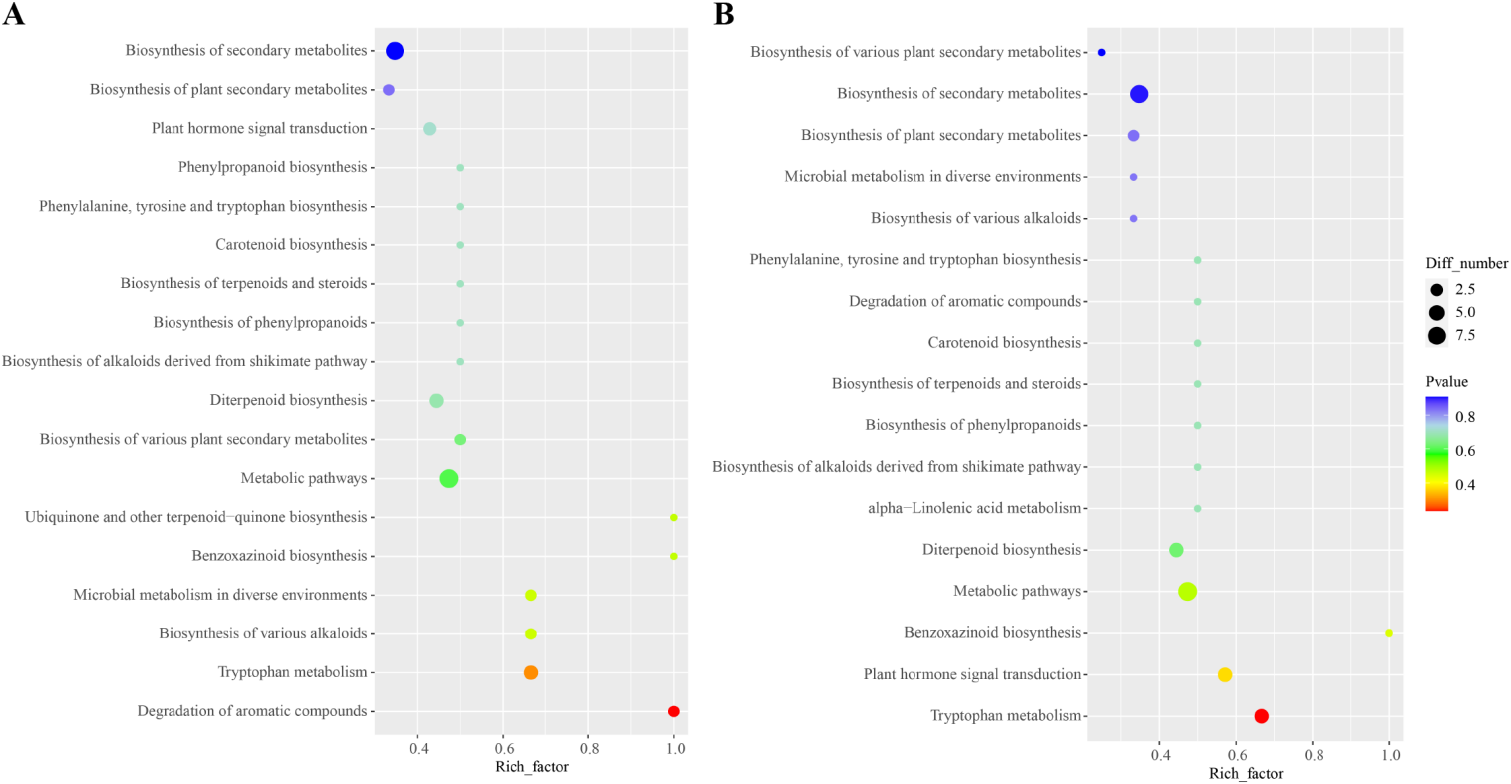
KEGG pathway analysis of DAMs. KEGG pathway analysis of DAMs in W4–20 vs. W4-10 **(A)** and W4–20 vs. W4-15 **(B)**.

For the W4–20 vs. W4–10 comparison (**Figure 4A**), a total of 18 KEGG pathways were enriched. Among them, “Degradation of aromatic compounds” and “Tryptophan metabolism” pathway were highly enriched. For the W4–20 vs. W4–15 comparison (**Figure 4B**), 17 KEGG pathways were identified. Notably, “Tryptophan metabolism” and “Benzoxazinoid biosynthesis” were significantly enriched in both the W4–20 vs. W4–15 and W4–20 vs. W4–10 comparison. In addition, “Plant hormone signal transduction” and “Diterpenoid biosynthesis” were also highly enriched in these comparisons.

#### 3.3.4 Correlation analysis of phytohormones and bioactive compounds

**Figure 5:**
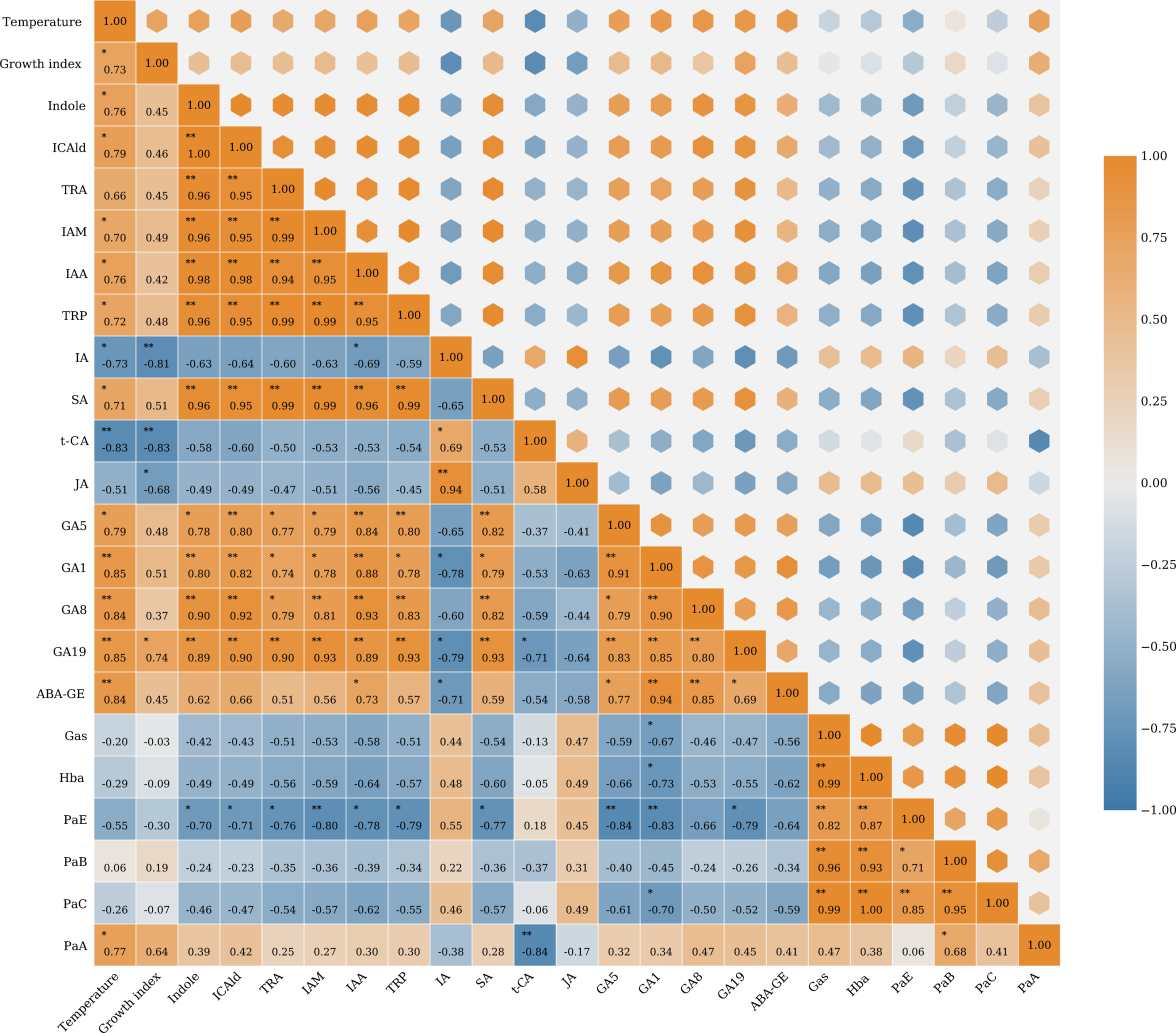
Correlation heatmap of temperature, growth index, DAMs and bioactive compounds. *: p<0.05; **: p<0.01.

To further examine the relationships between plant hormones and major bioactive compounds under different temperatures, a correlation analysis was conducted based on the quantified metabolite data of DAMs and the contents of bioactive compounds. As shown in **Figure 5**, growth index, parishin A (PaA) and most DAMs were significantly positively correlated with temperature. The growth index had a significantly positive correlation with GA19, while it showed a significantly negative correlation with IA, t-CA and JA.

The correlation matrix identified two major metabolite clusters that showed contrasting association patterns. The first cluster mainly included auxin-related metabolites, such as Indole, ICAld, TRA, IAM, IAA, and TRP, along with several gibberellin-related compounds, including GA1, GA5, GA8, and GA19. Strong positive correlations were observed within this cluster. In particular, intermediates in the indole pathway were strongly positively correlated with IAA and TRP, and they were also positively associated with most gibberellins. In contrast, the second cluster mainly comprised the major bioactive compounds, including gastrodin (Gas), p-hydroxybenzyl alcohol (Hba), parishin E (PaE), parishin B (PaB), parishin C (PaC) and parishin A. These compounds were positively correlated with each other, suggesting coordinated accumulation of the main medicinal constituents. However, most auxin- and gibberellin-related metabolites were negatively correlated with these major bioactive compounds. Among them, parishin E showed particularly strong negative correlations with indole, ICAld, TRA, IAM, IAA, TRP, SA, and several gibberellin-related metabolites. P-hydroxybenzyl alcohol, parishin A and parishin C also showed broadly negative correlations with auxin- and gibberellin-related metabolites. Overall, the correlation analysis suggested that phytohormones and bioactive compounds showed contrasting patterns across temperature treatments.

## 4 Discussion

### 4.1 Effects of temperature on biomass

*G. elata* f. *glauca* is primarily adapted to habitats above 1,500 m in altitude. For example, Xiaocaoba Town in Zhaotong, Yunnan Province, one of its major production areas, is located at approximately 1,710 m and has an annual mean temperature of 15.5 °C (Li et al., 2025). In the present study, tuber growth was markedly retarded at 4 °C. Given that *G. elata* f. *glauca* is fully mycoheterotrophic and relies on *Armillaria*-mediated carbon and nutrient transfer at the symbiotic interface, impaired fungal development under 4 °C would directly translate into insufficient nutrient supply for tuber enlargement. Previous studies have shown that *Armillaria* mycelial growth generally requires ambient temperatures above 6–8 °C, with an optimum of 20–22 °C (Lech et al., 2023). Moreover, vigorously growing and more highly branched mycelia are also more favorable for increasing *G. elata* yield (Wang et al., 2023). As temperature increased, the growth index of *G. elata* f. *glauca* rose steadily and reached a maximum at 20 °C. This result is consistent with the findings on semi-wild cultivation across different altitudes (Li et al., 2020). Their results indicated that areas at approximately 1,500 m with monthly mean temperatures ranging from 10.2 °C to 21.9 °C between February and October represent the optimal growth zone for *G. elata* f. *glauca*. However, when the cultivation temperature was increased to 25 °C, the growth index decreased markedly. This pattern suggests that moderate warming promotes dormancy release and the transition to active growth, thereby accelerating cellular metabolism and dry matter accumulation, whereas excessively high temperature inhibits growth and may even lead to tuber rot (Luo et al., 2010; Wang et al., 2022).

### 4.2 Effects of temperature on bioactive compounds

Temperature is one of the most important environmental factors regulating the biosynthesis and accumulation of bioactive constituents in medicinal plants by potently regulating secondary metabolic pathways (Zheng et al., 2025). Zhu reported that temperature had the strongest correlation with both the extract yield of *G. elata* and the contents of its major active constituents. Specifically, gastrodin, p-hydroxybenzyl alcohol, and the total amount of parishin A, B, C, and E were significantly and positively correlated with annual mean temperature (Zhu et al., 2023). Meanwhile, the accumulation of secondary metabolites often reflects the physiological responses and adaptive strategies of plants under different temperature conditions. In the study on *Dendrobium cariniferum* Rchb. f, Lin observed that at 23 ± 2 °C, substantial nutrients were preferentially allocated to vegetative growth, resulting in relatively lower accumulation of bioactive compounds, whereas at 25 ± 2 °C, secondary metabolite contents were significantly higher than those at other tested temperatures (Lin et al., 2020). In contrast, low-temperature stress enhanced the accumulation of mannose and total flavonoids in *Dendrobium huoshanense*, thereby providing cold tolerance (Wu et al., 2023).

The present results demonstrated that gastrodin, p-hydroxybenzyl alcohol, and parishin A, B, and C reached relatively high levels at 15 °C, whereas their contents generally declined at 20-25 °C. In contrast, parishin E maintained high accumulation at 25 °C, indicating clear compound-specific responses to temperature. These findings suggest that 15 °C may provide a favorable and stable metabolic environment for the phenylpropanoid pathway, as well as for downstream glycosylation and esterification modifications, thereby promoting the synthesis of simple phenolic precursors and complex phenolic glycosides. At higher temperatures, however, metabolic resources may be preferentially directed toward basal physiological maintenance or stress responses, reducing the biosynthetic efficiency of these secondary metabolites. The preferential accumulation of parishin E under high temperature and at the late growth stage further suggests differences in metabolic regulation and structural stability among parishin compounds. This pattern may be linked to distinct expression profiles of glycosyltransferase genes involved in parishin E biosynthesis (Wang et al., 2025).

### 4.3 Effects of temperature on plant hormones

Plant hormones play pivotal roles in coordinating plant growth, development, and environmental stress responses (Fàbregas et al., 2026). In this study, targeted metabolomics was employed to quantify eight major classes of plant hormones, along with their precursors and metabolites. The results showed that W4–20 vs. W4–10 and W4–20 vs. W4–15 comparisons exhibited a larger number of DAMs, most of which were upregulated. While W4–10 vs. W4–15 comparison exhibited the fewest DAMs, all of which were downregulated. This indicates that elevated temperature promotes the accumulation of hormone-related metabolites to support plant growth and development, whereas lower temperature appears to constrain hormone-associated metabolic processes, possibly to prioritize other physiological pathways. Notably, the upregulated DAMs in W4–20 vs. W4–10 and W4–20 vs. W4–15 were identical, and only one downregulated metabolite differed between the two comparisons. These findings indicate that the metabolic response induced at 20 °C was reproducible and directionally consistent.

Auxin regulates plant cell division, apical dominance, and root elongation, and its biosynthesis and signal transduction are influenced by environmental cues (Guo et al., 2021; Vaddepalli et al., 2021; Gabarain et al., 2025). Gibberellins promote stem elongation, seed germination, and flowering (Zhang et al., 2023; Xu et al., 2025; Xian et al., 2024). Moreover, auxin and gibberellins act synergistically with other hormones to coordinate carbon allocation, thereby balancing plant growth and defense (Mäkilä et al., 2023; Jin et al., 2023). In our research, compared with the 10 °C and 15 °C treatments, the upregulated DAMs at 20 °C were mainly auxin- and gibberellin-related compounds. KEGG pathway enrichment analysis revealed that these DAMs were primarily enriched in “Tryptophan metabolism”, “Benzoxazinoid biosynthesis”, “Degradation of aromatic compounds”, and “Plant hormone signal transduction”. These results suggest that elevating cultivation temperature to 20 °C may enhance the production of tryptophan–indole intermediates indicating a potential association with driving auxin biosynthesis and promoting tuber growth. Concurrently, enhanced diterpenoid biosynthesis and upregulation of gibberellins components may contribute to enhanced cellular vigor. This temperature-dependent regulation of auxin and gibberellins pathways is consistent with findings in *Arabidopsis thaliana*. Serivichyaswat reported that moderately elevated temperature promotes auxin biosynthesis and transport, thereby enhancing vascular bundle formation (Serivichyaswat et al., 2022). Similarly, high-temperature conditions can induce TCP transcription factor to upregulate gibberellins biosynthetic genes such as GA20ox1, thereby promoting cell expansion (Ferrero et al., 2019).

When cultivation temperature decreased from 15 °C to 10 °C, only two indole-related DAMs were downregulated in *G. elata* f. *glauca*. Previous studies in *A. thaliana* have shown that low temperature alters auxin content, distribution, and polar transport, thereby reducing meristem activity and slowing developmental progression (Zhu et al., 2015). Accordingly, *G. elata* f. *glauca* may adapt to low temperature by reducing reaction rates and metabolic fluxes, thereby suppressing the formation of tryptophan- and indole-related intermediates.

Indole is not only a key intermediate in the tryptophan-indole metabolic network but can also function as a volatile infochemical-like signal in defense responses, linking Ca^2+^ signaling with hormone modules such as JA and thereby reshaping downstream secondary metabolism and resistance (Erb et al., 2015). Indole-3-carboxaldehyde is a representative metabolite in the tryptophan-derived indole defense pathway, and its variation can reflect the activation level of immunity-related indole secondary-metabolic modules. It is often coupled with immune-hormone networks such as SA and JA, and can therefore be regarded as an output of the growth-defense balance (Böttcher et al., 2014). Together with the differential expression of other indole-branch metabolites and KEGG annotation results, these findings indicate that, during *G. elata* f. *glauca*’s adaptation to temperature, indole, indole-3-carboxaldehyde, and related indole metabolites may play important roles in tuber growth, secondary metabolite biosynthesis, and defense.

### 4.4 The influence of plant hormones on growth and bioactive compounds accumulation

Plant hormones play essential roles in regulating both growth and secondary metabolism in medicinal plants (Gasperini and Howe, 2024). In this study, correlation analysis further showed that temperature regulates the balance between growth and bioactive compound accumulation in *G. elata* f. *glauca* through plant hormones metabolism.

A major feature of the correlation matrix was the strong positive association between auxin-related metabolites and several gibberellin-related compounds. The higher abundance of these metabolites was consistent with the superior growth observed at 20 °C. Moreover, the growth index was positively correlated with temperature and with most auxin- and gibberellin-related metabolites. In contrast, the growth index was negatively correlated with IA and t-CA, suggesting that these metabolites may be less favorable for rapid growth. These findings suggest that elevated temperature promotes a hormone-related metabolic program that supports biomass accumulation. Previous studies also have shown that auxin and gibberellins promote tuber size in potato, root growth in *Panax ginseng*, and storage root development in sweet potato (Hong et al., 2021; Xue et al., 2024; Roumeliotis et al., 2023).

In contrast, gastrodin, p-hydroxybenzyl alcohol, and several parishin compounds formed a separate positively correlated part, indicating coordinated accumulation of the principal medicinal constituents. However, the growth index showed weak correlations with bioactive compounds, further indicating that rapid biomass accumulation was generally not accompanied by greater accumulation of the principal medicinal constituents. Furthermore, these bioactive compounds were generally negatively correlated with most auxin- and gibberellin-related metabolites. These contrasting patterns support a temperature-mediated trade-off between growth and medicinal quality in *G. elata* f. *glauca*. At 20 °C, increased auxin- and gibberellin-related metabolism coincided with enhanced growth, suggesting that metabolic resources were preferentially allocated to developmental processes. In contrast, 15 °C favored the accumulation of gastrodin and parishins, despite the lack of strong activation of growth-promoting hormone metabolism. Similarly, elevated gibberellin levels inhibit flavonoid biosynthesis while enhancing nitrogen metabolism in *Medicago truncatula* (Sun et al., 2021).

Notably, hormone-related differences between 10 °C and 15 °C were small. This suggests that the higher accumulation of bioactive compounds at 15 °C was not caused by strong hormonal induction. Instead, it may reflect a more moderate and balanced metabolic state. Such a state may favor carbon allocation to secondary metabolism. Jan Zrimec constructed a model of primary and secondary metabolism in potato to evaluate plant growth–defense trade-offs. They found that lower relative growth rate was associated with increased secondary metabolite levels, indicating a dynamic balance between biomass production and defense compounds (Zrimec et al., 2025).

Not all metabolites followed the dominant pattern. IA and JA showed correlation profiles that differed from those of the main auxin–gibberellin module, and parishin A also differed from the other parishin compounds. These results suggest that temperature-dependent regulation of medicinal compound accumulation is not uniform. Rather, it involves different contributions from specific hormone pathways and distinct regulation of individual phenolic derivatives.

## 5 Conclusion

In this study, we systematically quantified the growth, bioactive compounds accumulation, and targeted phytohormone profiles of *G. elata* f. *glauca* under different cultivation temperatures. These results showed that the growth index reached its maximum at 20 °C. In contrast, gastrodin, p-hydroxybenzyl alcohol, and parishin A, B, and C accumulated to their highest levels at 15 °C, whereas parishin E showed preferential accumulation at 25 °C. Targeted metabolomics indicated that temperature effects tryptophan metabolism and diterpenoid biosynthesis, together with shifts in auxin- and gibberellin-related metabolites. At 20 °C, increases in auxin- and gibberellin-related metabolites were accompanied by biomass accumulation but lower levels of bioactive compounds. By contrast, 15 °C was associated with a more balanced hormone-related metabolic profile and greater accumulation of pharmacologically active compounds in *G. elata* f. *glauca.* The correlation analysis further indicated that temperature significantly affected biomass accumulation, bioactive compounds accumulation through hormone metabolism. The growth index had a significantly positive correlation with GA19, while it showed a significantly negative correlation with IA, t-CA and JA. However, most auxin- and gibberellin-related metabolites were negatively correlated with major bioactive compounds. In addition, although coordinated variation in growth, phytohormones, and bioactive constituents was observed, the causal relationships among these processes remain unclear and require further validation through gene expression analysis, enzyme activity assays, and other functional experiments. These findings provide useful evidence for optimizing cultivation conditions for standardized high-altitude production of *G. elata* f. *glauca*. They also lay a foundation for future mechanistic studies on the metabolic regulation of characteristic quality formation.

## Data Availability

The raw data supporting the conclusions of this article will be made available by the authors, without undue reservation.
